# Economic returns in forming stable R&D networks

**DOI:** 10.1186/s40064-016-3260-8

**Published:** 2016-09-15

**Authors:** Mohamad Alghamdi

**Affiliations:** Department of Mathematics, College of Sciences, King Saud University, P.O. Box 2455, Riyadh, 11451 Saudi Arabia

**Keywords:** Stable networks, Network size, Equilibria, Profit maximization

## Abstract

The aim of this paper is to study the individual and social benefits behind constructing stable R&D networks. We find that the equilibrium outcomes of a stable network are related to the number of competitors. As they increase, the individual outcomes and the total welfare decrease. This implies that in the individual and social perspectives, small stable networks are more desirable than the large ones. Furthermore, when comparing the stability of the components of a network with a complete network, we conclude two main observations. The first observation shows that the stability of the components of a network does not necessarily guarantee a stable overall network. The second observation suggests that firms prefer to be part of a complete network rather than part of a stable component of a network. This preference depends on the profit of firms where it is maximized when firms are belong to the complete network.

## Introduction

In the empirical R&D literature, several studies found that the substantial increase of the R&D agreements in many industrial sectors had a role in maintaining firms in the marketplace (e.g., Hagedoorn et al. 2000; Hagedoorn and van Ekert 2002; Belderbos et al. 2004). Other studies discussed the cooperation of firms in R&D under a network concept (e.g., Fritsch and Lukas 2001; Okamuro 2007; Miotti and Sachwald 2003; Tomasello et al. 2013). The focus of those papers was on determining the structural changes of the network over time. The main conclusion for different samples of data reported in those papers demonstrates the intensity of R&D agreements. This interesting observation means that the cooperation exhibit characteristic features of complex networks (Ahuja 2000; Stuart 2000; Verspagen and Duysters 2004; Tomasello et al. 2013). The authors found that highly connected firms (firms with many links) have a role in constructing such complexity.

For the purpose of the current study, the most important conclusion of those empirical works lies in continuing the overall structure of the cooperation network over time. In a sense that the complex R&D network almost becomes a stable network. This leads us to investigate theoretically the stability of the R&D network. In the theoretical R&D literature, the most common stable R&D structure is a complete network (Goyal and Moraga-Gonzalez 2001; Goyal and Joshi 2003; Song and Vannetelbosch 2007; Deroian 2008; Westbrock 2010; Zu et al. 2011; Konig et al. 2012; Zirulia 2012). This means that the current paper mainly focuses on the complete networks.

We consider a network game for firms conduct R&D to reduce the cost of the production. The model structure is based on Goyal and Moraga-Gonzalez (2001), and it can be briefly described as follows. The structure that displays firms cooperate in R&D can be described as an R&D network where the players (firms) are represented by nodes and the R&D partnerships (cooperation) are represented by links. The model consists of three stages: network formation, R&D investment (effort) and market competition. The marginal cost of the production decreases with increasing the individual effort and effort of other firms in the network. Specifically, if any two firms are linked, the spillover is set one; otherwise there is a free spillover less than one (the ability to take advantage of partners’ R&D investment).

Specifically, Goyal and Moraga-Gonzalez readdressed the R&D model as R&D network where R&D effort (investment) is endogenous. They ignored the cost of the cooperation and considered Cournot competition for symmetric and asymmetric networks with independent and homogeneous goods. They found that for independent goods, the complete network (each two nodes in the network are linked) is uniquely stable network. Also, they stated that the complete network is stable if goods are homogeneous whatever the value of the R&D spillover.

There are many papers based on Goyal and Moraga-Gonzalez model (e.g., Song and Vannetelbosch 2007; Zirulia 2012; Zu et al. 2011). Song and Vannetelbosch (2007) studied the case when there are three firms belonging to different countries and producing homogeneous goods. For asymmetric R&D networks with and without government subsidies, they found that the complete network is stable. Zu et al. (2011) focused on the stability and efficiency of R&D networks for three international firms. They also found that the complete network is stable. Moreover, Zirulia (2012) extended Goyal and Moraga-Gonzalez model by linking the R&D spillover to technological heterogeneity. They assumed that there are two technological groups where firms in the same group have the same technology; whereas firms from different groups have different technologies. They stated that the complete network is stable. In addition, Goyal and Joshi (2003) studied cooperation of firms under different network model. In their model, the effects of link formation are exogenous, in particular the marginal cost decreases with the number of the R&D partners. They considered this set for Cournot and Bertrand competition where the cost of the cooperation is considered. They found that in the case of Cournot competition that the complete network is uniquely stable network.

The current paper contributes to the theoretical R&D literature through studying the interests of constructing the stable architecture of R&D cooperation. In particular, this paper carries two aims. The first aim is to investigate theoretically the relationship between the economic returns on the outcomes and the expansion of the stable network. The second aim is to study the stability of the components of R&D networks and compare the outcomes with those resulting from small stable networks. According to Goyal and Moraga-Gonzalez, if goods are independent, the outcomes are positively affected by the number of cooperative links. This indicates that the expansion of the R&D network always improves the equilibrium outcomes. Because of that, we do our study in this paper for homogeneous goods.

For the first aim, we assume that the network size increases and with each newcomer, firms form a complete network (a stable network).^1^ We investigate how the number of firms affects the private incentives to form intense collaboration. The results suggest that the relationship between the equilibrium outcomes of the complete network and the number of firms is monotonic. For the individuals, the higher size of a stable network produces smaller outcomes. This means the R&D effort and the quantity of the production decrease with growing the size of the stable network. For the profits of firms, while Goyal and Moraga-Gonzalez stressed the value of the R&D agreements, increase size of the complete network reduces the firms’ incentives to form the cooperation. That is, if the existing and newcomer firms work to build a complete network, their profits will decrease. This result may reflect the reality of the real-world cooperation between firms where it is impossible to be described the real network as a complete network.

Moreover, there are two other observations on the profit of firms in a growing complete network. The first observation suggests that the reduction in the profit is not fixed despite of the regular growth in the network. The second observation concerns the outcomes in a large complete network. As the number of firms increases, it is expected that the profits reach the lower level. Also, the growth of the complete network has a negative impact on the industry profit and overall welfare. However, for the consumer surplus, the expansion the complete network generates high outcomes.

For the second aim of this paper, we assume a network consists of several complete components and a complete network. Firstly, the stability of components does not generate a stable overall network. When comparing the outcomes of the stable components of a network with the outcomes of the complete network consisted of the same size of each component, we find that the individual outcomes of the complete components are smaller than the outcomes of the complete network. Secondly, comparing the outcomes of the complete giant component with the outcomes of other components of a network shows that the individual outcomes are higher in the giant component.

The paper is structured as follows. In the second section, we provide foundations in the social network and microeconomics and we introduce the Goyal and Moraga-Gonzalez model. In the third section, we present our outcomes. In the fourth section, we conclude our study.

## Background

### Network

A **network** is formed by a set of vertices (nodes) and a set of edges (links) connecting these vertices (Newman 2003; Jackson 2008). We define *N* as a set of all vertices labeled by letters $i,j,k,\ldots$ where $|N|=n$ and $E=\left\{ ij, jk, \ldots \right\}$ is a set of all edges in the network where $|E|=m$ is the number of links. Then *G*(*N*, *E*) denotes a network with nodes *N* and links *E*, and for simplicity the network is denoted by *G*. For the purpose of this article, we focus on undirected networks; meaning that each link between any two vertices runs in both directions (i.e., each two links *ij* and *ji* in *G* are the same). We also focus on simple networks that have neither parallel edges (edges that have the same end vertices) nor loops (edges where their start and end vertices are the same).

Nodes linked to node $i \in N$ is defined as a set of neighbors of that node: $N_i=\left\{ j \in N : ij \in E \right\}$. The length of the neighbors’ set of node *i* is a degree of that node. Thus, the degree of each node $i \in N$ is denoted by $deg(i)=|N_i|$ where $0 \le deg(i) \le n-1$. If *G* is a **complete network** (each two nodes in *G* are linked), then for any $i \in G$, $deg(i)=n-1$. Usually, the complete network consists of *n* nodes is denoted by $K_n$. An **empty network**$E_{n}$ is a graph consists of *n* nodes without links between them.

A subgraph $G^{'}(N^{'},E^{'})$ of the network *G*(*N*, *E*) is a graph such that $N^{'} \subseteq N$ and $E^{'} \subseteq E$. A **component** of the graph *G* is defined as a connected subgraphs where the largest connected component is called the giant component.

### The model

The emphasis in this paper is on the linear-quadratic function of consumers given by Hackner (2000):

1 $$U=a \sum ^{n}_{i=1} q_i - \frac{1}{2} \left( \alpha \sum ^{n}_{i=1}q^{2}_{i} + 2 \lambda \sum _{j\ne i}q_i q_j\right) + I .$$

Here the demand parameters $a>0$ denotes the willingness of consumers to pay and $\alpha > 0$ is the diminishing marginal rate of consumption, while $q_i$ is the quantity consumed of good *i* and *I* measures the consumer’s consumption of all other products. The parameter $\lambda$ such that $-1\le \lambda \le 1$ captures the marginal rate of substitution between different products. In this paper, we consider homogeneous goods ($\lambda =1$) and to simplify the analysis, we assumed that $\alpha = 1$.

If the consumer buys $q_i$ of good *i* where *m* is a consumer’s income and $p_i$ is the price of good *i*, the money spent is $p_iq_i$ and the balance is $I=m-p_iq_i$. By substituting into (1), we determine the optimal consumption of good *i* by calculating $\frac{\partial U}{\partial q_i} = a - q_i - \lambda \sum _{j\ne i} q_j - p_i = 0$. This implies the inverse demand function for each good *i*

2 $$D_i^{-1} = p_i = a - q_i - \lambda \sum _{j\ne i} q_j ,\quad i=1,\ldots ,n .$$

The profit $\pi _i$ for firm *i* is

3 $$\pi _i=(p_i - c_i)q_i= \left( a - q_{i} - \lambda \sum ^{n}_{j \ne i}q_{j} - c_{i} \right) q_{i} ,$$

where $p_i$ is the price of good *i* produced by firm *i* and $c_i$ is the production cost.

The consumer surplus and total welfare are expressed as

4 $$CS= \frac{1}{2} \left( \sum ^{n}_{i=1} q_{i}\right) ^2 ,$$

5 $$TW= CS + \Pi ,$$

where $\Pi = \sum ^{n}_{i=1} \pi _i$ is the industry surplus.

### R&D network model

The focus of this paper is on Goyal and Moraga-Gonzalez model. In their model, if firms cooperate in R&D, they are linked in an undirected network and spillover is set at one where the cost of link formation is assumed to be negligible. If firms do not cooperate, they are not linked and there is a spillover ($\beta \in [0,1)$) between non-linked firms.

Goyal and Moraga-Gonzalez (2001) examined an oligopolistic market under Cournot competition with linear demand for symmetric and asymmetric networks. They focused on the impact of the cooperative links on R&D investment and on the incentives of firms to cooperate. Moreover, their study investigated the situations in which the conflict between the stability and efficiency of R&D networks occurs. For stability of the R&D network with homogeneous goods, they found that the complete network is a stable network.

1. Complete R&D network

In a complete network, each two firms are linked. We denote $K_{n}$ to the complete network consists of *n* firms. When we say the size of the complete network increases, we mean that the existing and new entering firms cooperate together to form a new complete network.

2. Stages of the model

In Goyal and Moraga-Gonzalez, firms strategically form bilateral collaborative links with other firms where the collaboration of firms is modeled as a three-stage game.

The first stage: Each firm chooses its research partners. Firms and the cooperative links together constitute a network of cooperation in R&D.

The second stage: Given the R&D network, each firm chooses the amounts of investment (effort) in R&D simultaneously and independently in order to reduce the cost of production.

The third stage: Given the R&D investments of each firm and the effective R&D effort (as determined by the R&D network), firms compete in the product market by setting quantities (Cournot competition) in order to maximize their profits.

3. Cost reduction

In Goyal and Moraga-Gonzalez, the effective R&D effort for each firm is defined by the following equation:

6 $$X_i=x_i+\sum _{j \in N_i} x_j + \beta \sum _{k \notin N_i} x_k, \quad i=1, \ldots , n ,$$

where $x_i$ denotes R&D effort of firm *i*, $N_i$ is the set of firms participating in a joint venture with firm *i* and $\beta \in [0,1)$ is an exogenous parameter that captures knowledge spillovers acquired from firms not engaged in a joint venture with firm *i*. The effective R&D effort reduces firm *i*’s marginal cost ($\overline{c}$) of production

7 $$c_i=\overline{c}- x_i - \sum _{j \in N_i} x_j - \beta \sum _{k \notin N_i} x_k, \quad i=1, \dots , n .$$

The effort is assumed to be costly and the function of the cost is quadratic, so that the cost of R&D is $\gamma x^{2}_{i}$, where $\gamma >0$ indicates the effectiveness of R&D expenditure (D’Aspremont and Jacquemin 1988). The profit $\pi _i$ for firm *i* is the difference between revenue and production cost minus the cost of R&D

8 $$\pi _{i} = \left( a - \sum ^{n}_{i = 1} q_i -\overline{c}+ x_i + \sum _{j \in N_i} x_j + \beta \sum _{k \notin N_i} x_k\right) q_i - \gamma x^{2}_{i}\quad i=1, \ldots , n ,$$

where the marginal cost satisfy $a > \overline{c}$.

4. Equilibria for complete networks

In a complete network, each two firms are linked, thus the effective effort function for each firm *i* becomes

9 $$X_i=x_i+\sum _{j \in N_i} x_j, \quad i=1, \ldots , n .$$

From the Eq. (8), we can find the best response function of R&D effort for each firm *i* by calculating the first order condition ($\frac{\partial \pi _i}{\partial x_i}=0$). By substituting them to each other, we find the equilibrium effort. Then, we go backward to find the other equilibria. In the “Appendix”, we list the equilibria under homogeneous Cournot competition [cited from Goyal and Moraga-Gonzalez (2001)].

5. Effectiveness $$\gamma$$

According to Goyal and Moraga-Gonzalez, if goods are homogeneous ($\lambda = 1$), then the effectiveness should satisfy

10 $$\gamma > max \{ n^2/(n+1)^2,a/4\overline{c} \} .$$

The condition shows that $\gamma = 1$ is sufficient for any network size *n*.^2^

6. Pairwise stability and efficiency

The study of R&D cooperation under the network game involves the concepts of pairwise stability and efficiency. The pairwise stability depends on firms’ profit functions and it is a necessary condition for strategic stability as shown in Jackson and Wolinsky (1996).

#### **Definition 1**

(*Pairwise stability*) For any network *G* to be stable, the following two conditions need to be satisfied for any two firms *i*, $j \in G$:

1. If $$ij \in G$$, $$\pi _i (G) \ge \pi _i(G-ij)$$ and $$\pi _j (G) \ge {\pi }_j(G-ij)$$,
2. If $ij \notin G$ and if $\pi _i (G) < \pi _i(G+ij)$, then $\pi _j (G) > \pi _j(G+ij)$,

$G-ij$ is the network resulting from deleting a link *ij* from the network *G* and *G* + *ij* is the network resulting from adding a link *ij* to the network *G*. From this definition, network *G* is stable if no firm can obtain higher profit from deleting one of its links; and any other link between two firms would benefit only one of them.

The definition of the efficiency of a network that is given as follows and is determined by the total welfare generated from that network.

#### **Definition 2**

(*Network efficiency*) Network *G* is said to be efficient if no other network $\acute{G}$ can be generated by adding or deleting links, such that $TW(\acute{G}) > TW(G)$.

## The stability of R&D networks

In this section, we discuss two issues. The first issue concerns the impact of the expansion of the stable network on the economic outcomes. The second issue concerns the stability of the components of a network. For the stability, we focus on the complete networks for two reasons. The first reason, this type of networks is stable, according to many studies (e.g., Song and Vannetelbosch 2007; Zirulia 2012; Zu et al. 2011; Goyal and Joshi 2003). The second reason, for the complete networks, we can derive general equations describe the economic variables from which we can conduct studies on their behavior.

### The impact of the growing size on the stability of the network

Generally, the growth of the stable network (i.e., the complete network) yields reduction in the equilibrium outcomes. For the individuals, the R&D effort, the production and the profit decrease with each newcomer firm into the network cooperating with all existing firms.

#### **Proposition 1**

*Given a complete network*$K_{n}$, *assume that the effectiveness*$\gamma$*satisfies * (10). *The R&D effort, quantity and profit of firms are monotonically decreasing functions with respect to the network size n*.

The proof is given in the “Appendix”.

**Fig. 1:**
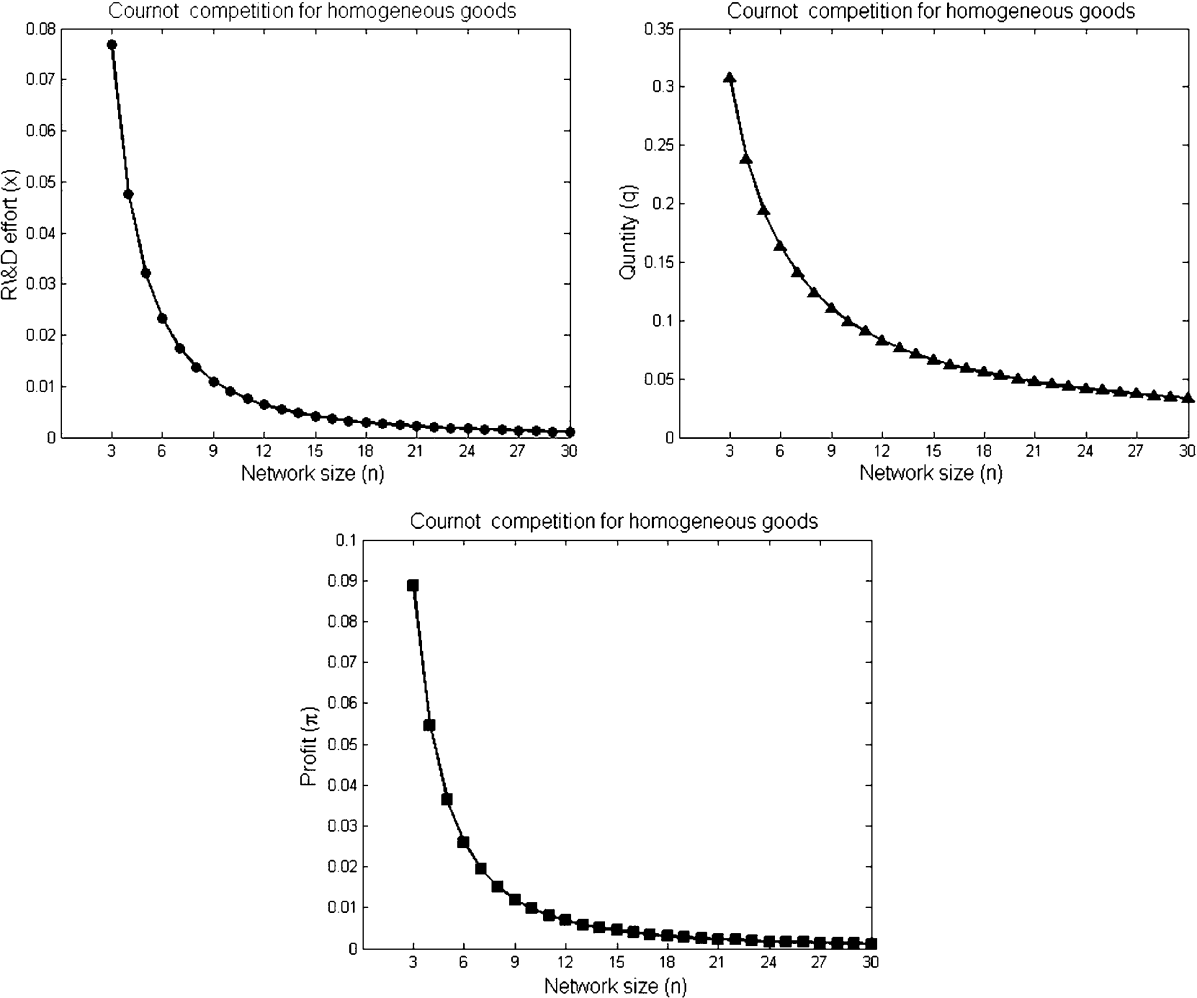
The R&D effort, quantity and profit of firms with increasing the complete network size. The parameters used to plot the figures are *a* = 2, $\overline{c} = 1$ and $\gamma = 1$ for $3\le n \le 30$

Concerning the profit of firms in the stable network $K_{n}$ (Proposition 1), we have two other results. The first result shows that the reduction in the profit by increasing the size of the stable network is not a constant amount (see Fig. 1). Meaning that for any *n*, $\pi _i(K_{n}) - \pi _i(K_{n+1}) \ne \pi _i(K_{n+1}) - \pi _i(K_{n+2})$.

#### **Corollary 1**

*Given complete networks*$K_{n}$*and*$K_{n+1}$, *assume that*$k_{n}$*and*$k_{n+1}$*are the number of links in that two networks, respectively. The profit in*$K_{n}$*and*$K_{n+1}$*can be written as*$\pi _i(K_{n+1}) = \pi _i(K_{n}) - \epsilon _{k_{n+1},k_{n}}$*such that*$\epsilon _{k_{n+1},k_{n}}$*is not a fixed amount among different stable networks.*

The second result relates to a large R&D network. The decrease in the individual profit in the stable network $K_{n}$ with increasing the firms indicates that in the large network, the profit is expected to be very small.

#### **Corollary 2**

*Given a large complete R&D network*$K_{n}$ ($n \rightarrow \infty$), *then*$\lim _{n\rightarrow \infty } \pi _i(K_{n}) = \delta$*where*$\delta > 0$*is very small.*

Previous results inferred directly from the Proposition 1 for this they do not need to prove. The following example shows these results with growing the size of the complete network.

#### *Example 1*

Let $K_{n}$ be a complete network with *n* firms. Assume that the size of $K_{n}$ increases from *n* = 3 to *n* = 30 where the resulting network with each new firm is a complete network. Figure 1 demonstrates the reduction of the R&D effort, quantity and profit with increasing the network size *n*.

The question that can be raised here is, with growing the size of a network, how fast profits vanish in an empty and a complete network? In other words, how the number of firms impact the profits when all firms share their knowledge compared to the case when each of them participate in R&D alone? Firstly, since the profit increases with cooperation, then the profit under the complete network is always more than under the empty network. Secondly, from Corollary 1, the gap between the profits under the two networks with increasing firms is not fixed.

Finally, when comparing the profits of firms under the complete and empty networks, the result shows that the gap decreases with a growing number of firms. The following proposition state this result and Example 2 provides more explanations.

#### **Proposition 2**

*Given networks*$K_{n}$*and*$E_{n}$, *assume that the effectiveness*$\gamma$*satisfies* (10). *The gap between profits of firms under two networks decreases with respect to the number of firmsn*.

The proof is given in the “Appendix”.

#### *Example 2*

**Fig. 2:**
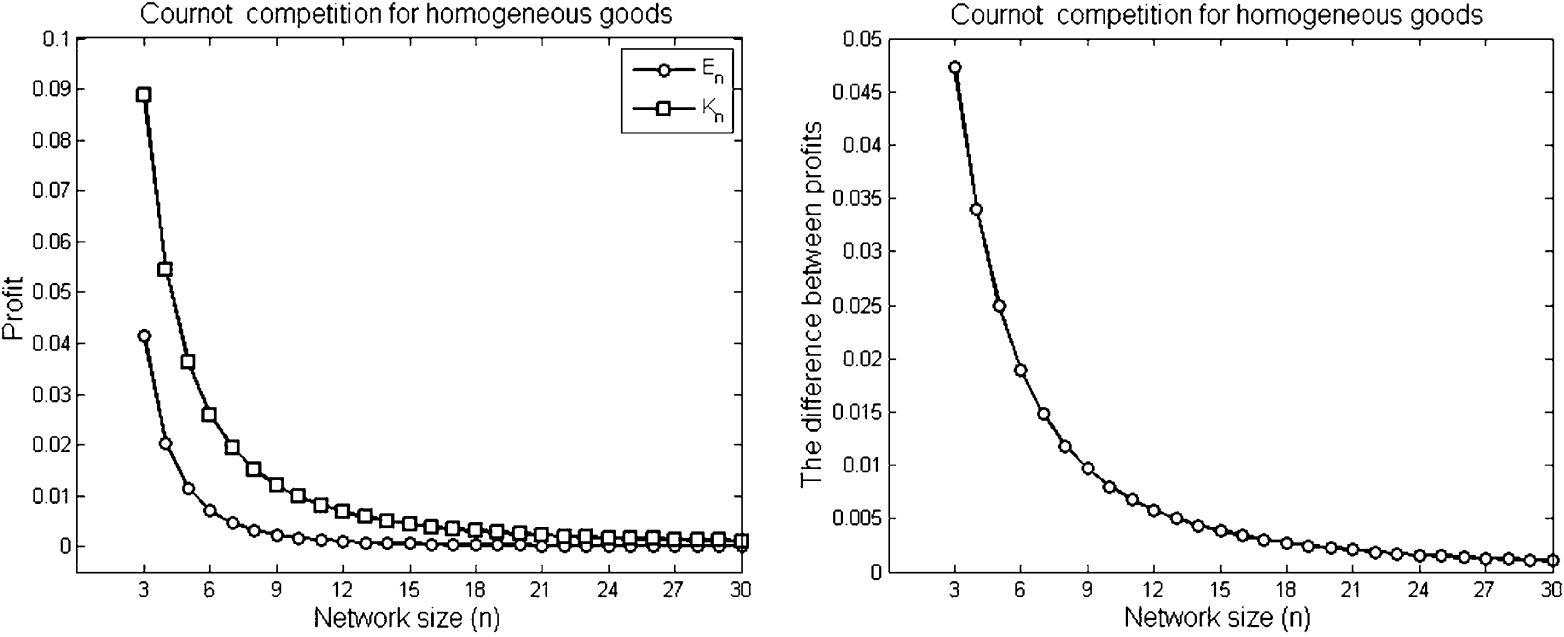
The profits of firms under the complete *K*_*n*_ and the empty network *E*_*n*_. The parameters used to plot the figures are *a* = 2, $\overline{c} = 1$ and $\gamma = 1$ for $3\le n \le 30$

For *n* firms, assume there are two different networks the complete and empty networks. Assume that the number of firms increases from *n* = 3 to *n* = 30 in both networks. Figure 2 shows the profits under the two networks with increasing *n*.

In the overall economic view, the growth of the population of the complete network has negative and positive impacts. The industry profit and overall welfare decrease in the number of firms in the complete network. However, the consumer surplus increases with growing the size of the complete network.

#### **Proposition 3**

*Given a complete network*$K_{n}$, *assume that the effectiveness*$\gamma$*satisfies the condition* (10). *With respect to the network sizen*,

1. *The industry profit and the total welfare are monotonically decreasing functions,*
2. *The consumer surplus is a monotonically increasing function.*

The proof is given in the “Appendix”.

The following example demonstrates the previous proposition.

#### *Example 3*

**Fig. 3:**
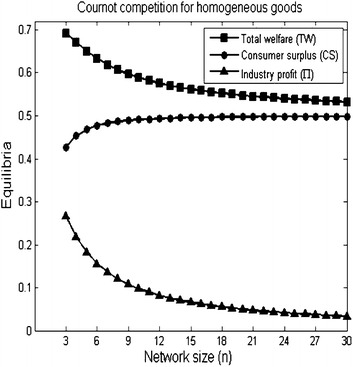
The R&D effort, quantity and profit of firms with increasing the complete network size. The parameters used to plot the figures are *a* = 2, $\overline{c} = 1$ and $\gamma = 1$ for $3\le n \le 30$

Let $K_{n}$ be a complete network with *n* firms. If the complete network size *n* increases, Fig. 3 shows a reduction in the outcomes of the industry profit and the total welfare and an increase in the consumer surplus outcome.

### Stability of components of networks

The stability of components of any network does not guarantee the stability of the overall network. For example, a network contains two complete components where each component contains three firms. Each component forms a stable network, but the resulting network of those components (a symmetric network with six firms) is not stable. This also occurs if the overall network consists of a giant component where the stability of that giant component does not mean that the overall network is stable.

#### **Proposition 4**

*Given a network G consists of n firms. If*$C_1, C_2, C_3, \ldots$*are stable components of G, the overall network G is not necessarily stable.*

#### **Corollary 3**

*Given a network G consists of n firms where GC is the giant component of G. If GC is stable, the overall network G is not necessarily stable.*

**Fig. 4:**
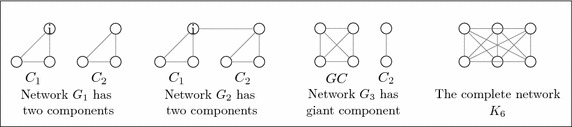
The figure shows four different networks *G*_1_, *G*_2_, *G*_3_ and *K*_6_

**Fig. 5:**
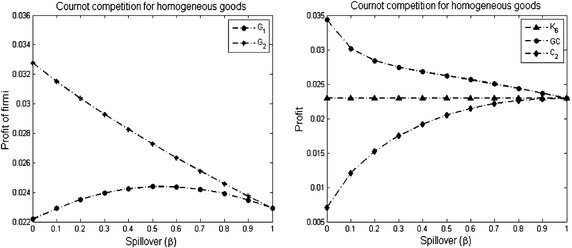
The individual outcomes in the networks given in Fig. 4. The figure on the *left* compares the profit of firm *i* in two different networks *G*_1_ and *G*_2_. The figure on the *right* compares the profit in the components *GC* and *C*_2_ and in the complete network *K*_6_. The parameters used to plot the figures are *a* = 2, $\overline{c} = 1$ and $\gamma = 2$

The first two networks in Fig. 4 consist of two complete components before and after connecting them i. e., $G_1$ and $G_2$ respectively. Figure 5 (left) compares the profit of firm *i* in those networks. Figure 5 (right) compares the profit of firms in the complete network $K_6$ with their profits in a network $G_3$ that consists of a giant component *GC* and another component *C*2.

#### *Example 4*

Figure 4 shows a network $G_1$ has two complete components, each of size 3 firms and a network $G_2$ consisted of two linked complete components. Also, the figure shows a network $G_3$ contains on a giant component and a complete network $K_6$.

1. The network $G_1$ has two components where each one forms a complete network. Consider one component of them, for example $C_1$. This component is stable since it forms a stable network, similarly for the component $C_2$. Thus, the network $G_1$ consists of two stable components. However, if we consider the two components together ($C_1$ and $C_2$ in the network $G_1$), the overall network is not stable because each firm seeks to have a higher profit which is acquired by forming new links. This result can be verified by comparing the profit of firm *i* in $C_1$ in the network $G_1$ (before creating a link with any firm in $C_2$) with its profit in the network $G_2$ (after creating a link with one firm in $C_2$). Figure 5 (left) shows that the profit of firm *i* in $G_1$ is smaller than in the network $G_2$. This suggests that the network that consists of several stable components is not stable.
2. The network $G_3$ has a giant component *GC* and another component $C_2$. The giant component is a stable network since it forms a complete network with four firms. However, the overall network $G_3$ is not stable. This can be shown by comparing the profit of firms in the components *GC* and $C_2$ and in the network $K_6$. From Fig. 5 (right), Firms in $C_2$ have the lowest profits. These profits increase when firms in that component cooperate with firms in the giant component.

According to Goyal and Moraga-Gonzalez (2001), the individual outcomes (the production quantity and the profit) increase with increasing the own cooperative links. This implies that the individual outcomes of the complete giant component of the network *G* are higher than the outcomes of any component contained in *G*.

#### **Proposition 5**

*Given a network G consists of n firms where GC is the giant component. If GC forms a complete structure, then for any component C of G, we find*$q^{*}(GC) > q^{*}(C)$*and*$\pi ^{*}(GC) > \pi ^{*}(C)$.

Example 5 (Item 2) shows the previous result. Figure 7 compares the quantity and the profit in those two components. The result indicates that the outcomes in the giant component are higher than in the component $C_2$.

Now, assume a network consisted of complete components (stable components) like network $G_1$ in Fig. 7. Assume each complete component has *n* firms and let $K_n$ be a complete network has *n* firms. Proposition 6 compares the outcomes of the stable components with the outcomes of the complete network contained an exact number of firms in each component.

#### **Proposition 6**

*Given a network G consists of a set of complete components*$C_1, C_2, C_3, \ldots$*where each component is of size n. Let*$K_{n}$*be a complete network has n firms. With respect to the condition* (10), *the individual outcomes (quantity and profit) of each*$C_i$*are smaller than the outcomes of*$K_{n}$.

The previous results (from Proposition 4 to Proposition 6) do not need to prove because they naturally resulting from the link of the gain to the number collaborations (Goyal and Moraga-Gonzalez 2001).

#### *Example 5*

**Fig. 6:**
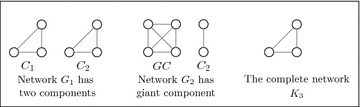
The figure shows three different networks: the network $G_1$ has two disconnected components, the network $G_2$ has giant component, and the complete network $K_3$

Figure 6 shows three different networks: the networks $G_1$ and $G_2$ given in Example 4 and the complete network $K_{3}$.

**Fig. 7:**
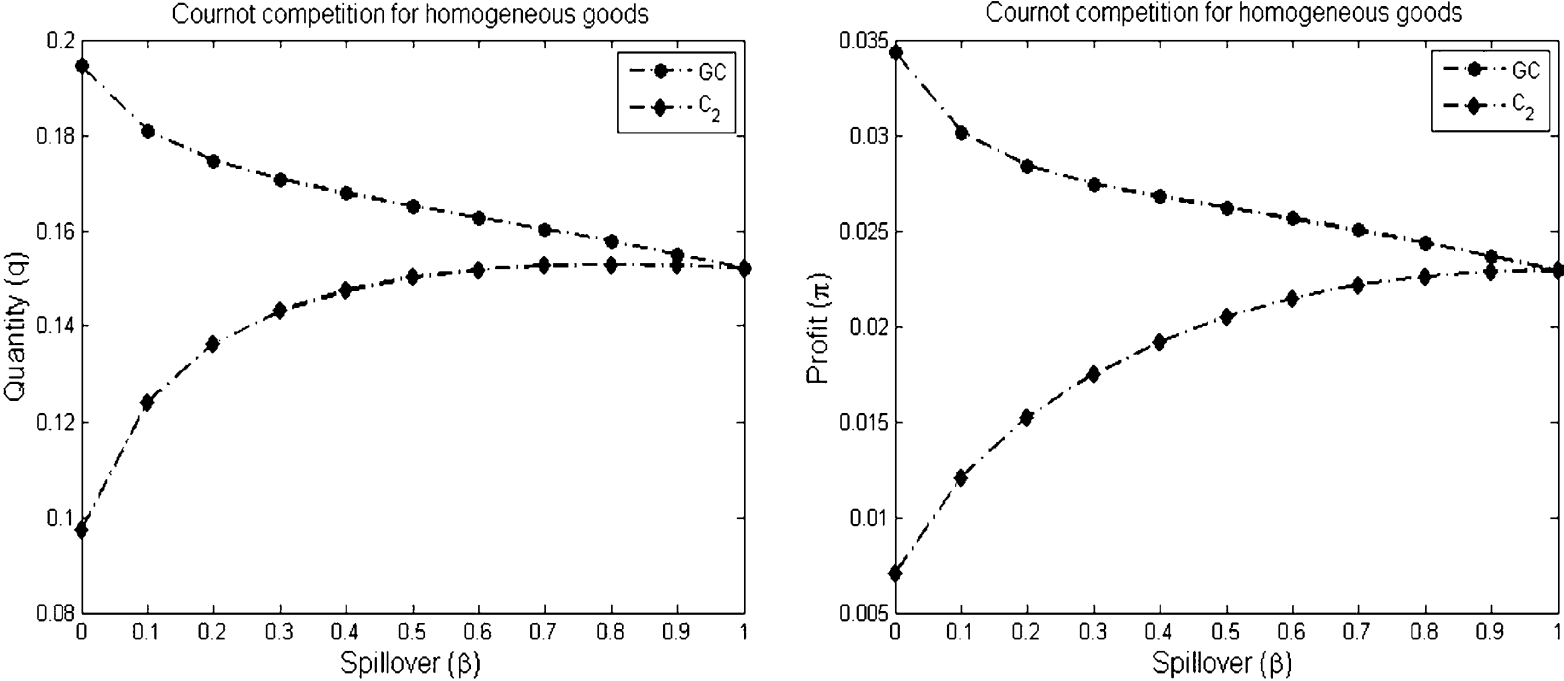
The individual outcomes in the network *G*_1_ consisted of a complete giant component *GC*. The figure compares the quantity and the profit in the components *GC* and *C*_2_. The parameters used to plot the figures are *a* = 2, $\overline{c} = 1$ and $\gamma = 2$

**Fig. 8:**
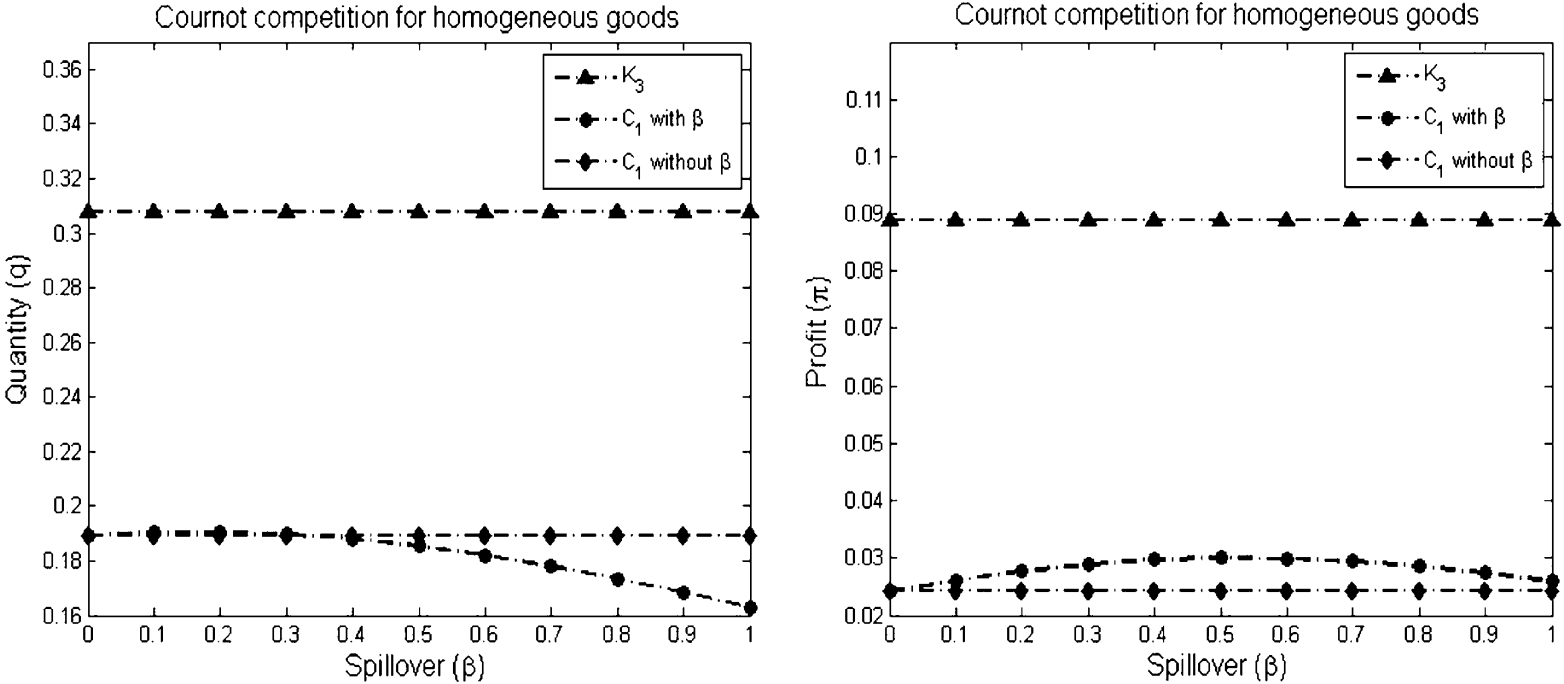
The individual outcomes in the complete network *K*_3_ and in the complete components *C*_1_ and *C*_2_. The size of each component is three firms as the size of *K*_3_. The parameters used to plot the figures are *a* = 2, $\overline{c} = 1$ and $\gamma = 1$

1. As shown in Fig. 7, the individual outcomes of the giant complete component *GC* is higher than the outcomes of the anther component $C_2$.
2. The individual outcomes (the quantity and the profit) of each complete component is lower than the outcomes in the complete network $K_{3}$ (see Fig. 8).

## Conclusion

The aim of this paper was to extend the analysis of the stability of R&D network formation with the intensity of competition. Under Cournot model with homogeneous products, the main results are summarized as follows.

Firstly, firms’ outcomes and the social welfare in stable networks are negatively related to the size of that network. This indicates that the improvement of the outcomes in the stable networks is acquired when firms belong to small networks. Secondly, examining the stability of the components and comparing the outcomes with those generating from stable networks showed that there is no relationship between the stability of a network and the stability of its components. Finally, the individual profits in a complete network are higher than their profits in small stable components.

## Appendix

The equilibria for the complete network $K_{n}$:

Effort: $$x^{*}=\frac{a-\overline{c}}{\gamma (n+1)^2-n}$$ .

Quantity: $$q^{*}=\frac{ \gamma (n+1) (a-\overline{c})}{\gamma (n+1)^2-n}$$ .

Profit: $$\pi ^{*}=\frac{ (\gamma ^2(n+1)^2 - 1) (a-\overline{c})^2}{(\gamma (n+1)^2-n)^2}$$ .

Consumer surplus: $$CS^{*}=\frac{n^2\gamma ^2(n+1)^2 (a-\overline{c})^2}{2(\gamma (n+1)^2-n)^2}$$ .

Total welfare: $$TW^{*}=\frac{n (\gamma ^2(n+2)(n+1)^2-2) (a-\overline{c})^2}{2(\gamma (n+1)^2-n)^2}$$ .

### *Proof of Proposition 1*

For simplicity, we prove the proposition for $(a-\overline{c}) = 1$.

1. The R&D effort

The equilibrium effort for the complete network consists of *n* firms is

$$x^{*}=\frac{a-\overline{c}}{\gamma (n+1)^2-n} .$$

We want to show that as *n* increases, the effort $x^{*}$ decreases. Calculate *dx* / *dn*, we have

$$\frac{d x}{d n} = \frac{-2\gamma (n+1) - 1}{(\gamma (n+1)^2 - n)^2} .$$

From the condition 10, we have $\frac{d x}{d n}<0$. This indicates that the R&D effort of firms in the network $K_{n}$ decreases as the size *n* increases.

2. The quantity

The equilibrium quantity of the complete network with *n* firms is

$$q^{*}=\frac{ \gamma (n+1) (a-\overline{c})}{\gamma (n+1)^2-n} .$$

Calculate *dq*/*dn*, we have

$$\frac{d q}{d n} = \frac{\gamma (\gamma (n+1)^2-n) - \gamma (n+1) (2 \gamma (n+1) - 1)}{(\gamma (n+1)^2 - n)^2} .$$

The terms $\gamma (n+1)^2-n$ and $2 \gamma (n+1)-1$ are positive (from the condition 10) and this yields $d q / d n < 0$. This implies that the production quantity in $K_{n}$ decreases with growing *n*.

3. The profit: The equilibrium profit for the complete network consists of *n* firms is

$$\pi ^{*}=\frac{ (\gamma ^2(n+1)^2 - 1) (a-\overline{c})^2}{(\gamma (n+1)^2-n)^2} .$$

By calculating $d \pi / d n$, we have

$$d \pi / d n = \frac{-2 \left [ (n+1)\gamma \left ( (n+1)^2\gamma ^2-(\gamma +2) \right ) +1 \right ]}{\left (\gamma (n+1)^2 - n \right )^3} .$$

Since $(n+1)^2\gamma ^2-(\gamma +2)>0$, then $d \pi / d n < 0$ which implies that the equilibrium profit decreases as the number of firms in $K_{n}$ increases.$\square$

### *Proof of Proposition 2*

The equilibrium profit for the complete and empty networks consists of *n* firms are

$$\begin{aligned} \pi ^{*} (K_n)&= \frac{ \left( \gamma ^2(n+1)^2 - 1\right) (a-\overline{c})^2}{\left( \gamma (n+1)^2-n\right) ^2} ,\\ \pi ^{*} (E_n)&= \frac{ \left( \gamma ^2(n+1)^2 - n^2\right) (a-\overline{c})^2}{\left( \gamma (n+1)^2-n\right) ^2} . \end{aligned}$$

The difference between the two profits is

$$\pi ^{*}_d=\frac{ (n^2-1) (a-\overline{c})^2}{(\gamma (n+1)^2-n)^2} .$$

Let $(a-\overline{c})=1$. By calculating $d \pi ^{*}_d / d n$, we have

$$\frac{2\left( \gamma (n+1)^2 (2-n) - 1 \right) }{\left[ \gamma (n+1)^2 - n\right] ^3} .$$

Since $\gamma (n+1)^2 (2-n) - 1<0$, then $d \pi ^{*}_d / d n < 0$. This implies that the difference between equilibrium profits under complete and empty networks decreases as the number of firms increases. $\square$

### *Proof of Proposition 3*

For simplicity, we prove the results by assuming $(a-\overline{c}) = 1$.

1. The consumer surplus

The consumer surplus for the complete network consists of *n* firms is

$$CS^{*}=\frac{n^2\gamma ^2(n+1)^2 (a-\overline{c})^2}{2\left( \gamma (n+1)^2-n\right) ^2} .$$

We want to show that $CS^{*}$ is an increasing function with *n*. From *dCS*/*dn*, we have

$$\frac{d CS}{d n} = \frac{n\gamma ^2 (n+1) \left( \gamma (n+1)^2 - n^2\right) }{\left( \gamma (n+1)^2 - n\right) ^3} > 0 .$$

This indicates that the consumer surplus increases as the complete network size *n* increases.

2. The industry profit

The proof of this item is straightforward from the equilibrium profit in Proposition 1.

3. The total welfare

The total welfare for $K_{n}$ is

$$TW^{*}=\frac{n \left( \gamma ^2(n+2)(n+1)^2-2\right) (a-\overline{c})^2}{2\left( \gamma (n+1)^2-n\right) ^2} .$$

From *dTW*/*dn*, we have

$$\begin{aligned} \frac{d TW}{d n} = \frac{-2 (n+1) \left[ 2n(n+1)^2(n+2) \gamma ^3 - (n^3+5n^2+6n+1)\gamma ^2-4n\gamma + 1 \right] }{2 (\gamma (n+1)^2 - n)^3} . \end{aligned}$$

For each network size *n* where the effectiveness $\gamma$ satisfies the condition (10), the expression inside the square bracket in the numerator is positive. This implies that $d TW / d n < 0$ which means the total welfare decreases with growing the complete network size. $\square$

## Footnotes

1 When we say size of the network, we mean the number of firms in the network.

2 To have suitable values of the effectiveness, the effort and cost functions should be non-negative and the second order condition for maximizing profit function ($\frac{\partial ^2 \pi }{\partial x^2}<0$) should be satisfied (Goyal and Moraga-Gonzalez 2001).
